# A Genome-Wide Longitudinal Transcriptome Analysis of the Aging Model *Podospora anserine*

**DOI:** 10.1371/journal.pone.0083109

**Published:** 2013-12-20

**Authors:** Oliver Philipp, Andrea Hamann, Jörg Servos, Alexandra Werner, Ina Koch, Heinz D. Osiewacz

**Affiliations:** 1 Molecular Developmental Biology, Institute of Molecular Biosciences, Faculty for Biosciences & Cluster of Excellence ‘Macromolecular Complexes’, Johann Wolfgang Goethe University, Frankfurt am Main, Germany; 2 Molecular Bioinformatics, Institute of Computer Science, Faculty of Computer Science and Mathematics & Cluster of Excellence ‘Macromolecular Complexes’, Johann Wolfgang Goethe University, Frankfurt am Main, Germany; Universidade de Sao Paulo, Brazil

## Abstract

Aging of biological systems is controlled by various processes which have a potential impact on gene expression. Here we report a genome-wide transcriptome analysis of the fungal aging model *Podospora anserina*. Total RNA of three individuals of defined age were pooled and analyzed by SuperSAGE (serial analysis of gene expression). A bioinformatics analysis identified different molecular pathways to be affected during aging. While the abundance of transcripts linked to ribosomes and to the proteasome quality control system were found to decrease during aging, those associated with autophagy increase, suggesting that autophagy may act as a compensatory quality control pathway. Transcript profiles associated with the energy metabolism including mitochondrial functions were identified to fluctuate during aging. Comparison of wild-type transcripts, which are continuously down-regulated during aging, with those down-regulated in the long-lived, copper-uptake mutant grisea, validated the relevance of age-related changes in cellular copper metabolism. Overall, we (i) present a unique age-related data set of a longitudinal study of the experimental aging model *P. anserina* which represents a reference resource for future investigations in a variety of organisms, (ii) suggest autophagy to be a key quality control pathway that becomes active once other pathways fail, and (iii) present testable predictions for subsequent experimental investigations.

## Introduction

Biological aging is a complex process leading to physiological impairments, the degeneration of cellular and organ functions, the development of disease and finally death of the system [1]–[3]. The underlying molecular mechanisms are multifactorial and only partially defined. It is clear that aging is accompanied by changes in gene expression. The available data, however, are mainly derived from single gene analyses and the comparison of only a few age stages (e.g., young vs. old). The comprehensive and systematic analyses of changes over the lifetime of individuals can identify new key pathways and regulatory circuits involved in aging and lifespan control and can open the field for the development of strategies to intervene into aging and age-related diseases (e.g., cancer, dementia, Parkinson’s disease, cardiovascular impairments). Nowadays, the availability of efficient high-throughput techniques makes such studies possible, in particular when the study is performed with experimentally accessible short-lived systems.

*Podospora anserina* is such a system [4]–[6]. In contrast to most filamentous fungi this ascomycete is characterized by a well-defined aging process that is under the control of genetic and environmental traits. After germination of an ascospore, a mycelium develops which grows at the periphery until it reaches a phase where the growth rate first decreases until it comes to a complete growth stop [7]. Finally, the hyphal tips burst and die. This process occurs under nutrient-replete growth conditions and thus clearly differs from those described as ‘aging’ in fungi grown under nutrient starvation [8] and as ‘chronological aging’ in the yeast *Saccharomyces cerevisiae* [9], [10]. In *P. anserina*, various molecular pathways have been identified to be involved in the control of aging and development [11]–[13]. The lifespan of the fungus is short (typically two to four weeks for wild-type strain ‘s’) and depends on the growth medium and on cultivation conditions [5], [14]. The vegetation body of *P. anserina* is simply consisting of branched filamentous cellsforming a mycelium. For sexual reproduction specialized organs, protoperithecia and spermogonia, are formed in dikaryotic as well as in monokaryotic strains. *P. anserina* is accessible to experimentation [4], [5]. Biomolecules like DNA, RNA or proteins as well as whole mitochondria can be isolated and analyzed from individuals of well-defined age [5]. The complete genome of *P. anserina* is sequenced and consists of about 36 MBp coding for more than 10,600 putative proteins [15], [16]. *P. anserina* can be genetically manipulated by classical genetic approaches and by genetic engineering [5], [17], [18].

Here we describe a genome-wide transcriptome profiling of three *P. anserina* individuals from which total RNA was isolated after 6, 9, 10, 11, 12, 13 and 14 days of cultivation. Quantitative transcript profiles were generated by serial analysis of gene expression (SuperSAGE) and analyzed by bioinformatical and statistical approaches [19]–[21]. Previously we used SuperSAGE successfully to characterize the transcriptome of a specific long-lived mutant of *P. anserina* and compared it to the transcriptome of the wild type. Validation by qRT-PCR demonstrated the reliability of this method [22]. The data of the current longitudinal study, in which RNA was isolated from the same fungal individuals after a defined period of growth and subjected to a genome-wide SuperSAGE analyses, identified autophagy as a quality control pathway up-regulated late in the life of *P. anserina* at a time when transcripts, encoding components of other pathways (e.g., proteasome), are down-regulated.

## Materials and Methods

### *Podospora anserina* Strains and Cultivation

For all experiments, three independent monokaryotic spore isolates (mating type minus) of the wild-type strain ‘s’ [7] were used. Cultivation was essentially performed as described previously [23]. Briefly, single ascospores were germinated for 2 days on germination medium. Pieces of mycelium of this two day old culture were either directly transferred to a fresh PASM [24] plate overlaid with a cellophane sheet or, in order to generate strains of older age, to solid PASM medium and incubated under permanent light at 27°C. After 5, 6, 7, 8, 9, and 10 days, respectively, pieces from the growth front of the latter cultures were transferred to a fresh PASM plate (overlaid with a cellophane sheet). After two days of growth, the mycelium of the developed culture was transferred from the cellophane to liquid CM medium [25] and incubated for additional 2 days at 27°C under light and agitation. This last incubation step leads to the formation of enough mycelium (biomass) that, free of agar, can be easily harvested for the isolation of RNA. Following this regime, mycelium grown for a defined period of time (different age stages) of 6, 9, 10, 11, 12, 13, and 14 days, respectively, was available for isolation of total RNA. To make sure that all three isolates have a similar aging behaviour, the lifespan as period of linear growth on solid PASM medium was recorded. All isolates had a lifespan of 14 days, thus the oldest age stage (14 days) represents a senescent culture.

### Isolation of Total RNA

Total RNA was isolated using a CsCl density gradient as described previously [22].

### Quantitative Real-time PCR

Quantitative Real-time PCR (qRT-PCR) was performed as described in [22]. Primer sequences can be found in Table S1.

### SuperSAGE Analysis

A SuperSAGE analysis [19] was performed for each of the seven samples consisting of the pooled RNA of three genetically identical individuals as described above. Sequence tag identification and annotation, and basal statistics were performed by GenXPro (Frankfurt) as described in [22]. The raw data have been deposited in the European Bioinformatics Institute’s ArrayExpress public data repository (http://www.ebi.ac.uk/arrayexpress/) [26] and are available under accession number E-MTAB-2016.

### Data Preparation and Filtering

Absolute tag counts were corrected for experimental biases and normalized to tags per million (tpm) in order to compare the different samples (see: Text S1). The latter is necessary because each library (absolute number of all tag molecules per sample) can differ from each other due to experimental and biological fluctuations. Furthermore, in order to compare the *n* different expression profiles they have been standardized to a mean expression strength of zero and a standard deviation of one:
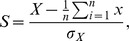
with *X* as the expression profile and σ*_X_* as its standard deviation. If analyses consider profiles in a Euclidean metric the regarding profiles were standardized, as well (e.g., for cluster analysis – see below). Expression profiles with more than 25% missing values were removed.

### Gene Ontology Analysis

At the time our transcriptome data became available, the genes in the genome of *P. anserina* were only poorly assigned to GO terms [21]. To extend the list of assignments for further analyses, we performed a BLAST [27] search of all 10,635 putative *P. anserina* genes and the proteins in the UniProt [28] data library adopting the information of other better annotated species. Subsequently, we assigned all GO terms of each hit with an E-value of ≤1e-20 to the corresponding gene of *P. anserina*. The complete annotation library is provided in Table S2.

The GO enrichment analysis was performed using the programming language R [29] by means of the R package GOstats [30] and an one-sided hyper geometric test (test for over-representation). GO terms were assumed to be statistically significantly enriched if the probability (p)-value was ≤0.01.

To identify and visualize possible links between individual GO terms deduced from the GO enrichment analyses, in selected cases, enrichment maps were generated [31] using the Cytoscape plugin [32]. Such a map is a graph-based representation which overcomes the redundancy problem in a GO enrichment analysis. In order to use the enrichment map plugin, a dedicated R script, providing the necessary interface, was developed. Basically, the enrichment analysis results preliminary generated by the GOstats package were adjusted and exported to a file containing the obligatory columns: GO identifiers, GO terms, enrichment p-values and phenotype (up- or down-regulated indicated by 1 or 0, respectively). Furthermore, a gmt-file was generated that contains all GO assignments, consisting of three columns: GO identifiers, GO descriptions, and all assigned gene accession numbers with the corresponding GO identifier. The R script is available upon request. In Cytoscape the default values for the enrichment map plugin were kept, only the p-value cutoff was set to 0.01, and a Jaccard coefficient of 0.25 as overlap coefficient was applied.

### Identification of Expression Profiles with Continuous Expression Tendencies

The gene expression profiles were searched for up- and down-regulated genes. For this purpose, each profile, i.e., the transcript abundance for each of the seven time points, was correlated with time (days 6, 9, 10, 11, 12, 13, 14) using the Pearson correlation coefficient. The threshold was set to 0.7. This threshold is accepted to indicate a strong (positive or negative) linear correlation of two variables or expression data, respectively [33], [34]. Thus, with a coefficient of ≤−0.7 the corresponding expression profiles show a decrease and profiles with a coefficient of ≥0.7 an increase during aging. We only considered profiles further if the p-value for differential expression of day 6 (first day in measurement) to day 14 (last day) is ≤1e-10.

### Significance Smoothing

The clustering algorithm we performed used a Euclidean metric. To compare the different patterns, it was necessary to standardize the expression profiles. A major drawback of standardization is that various information regarding variances and dispersion get lost. To avoid as much as possible an over-fitting and over-interpretation and to get rid of low abundant and/or biased expression profiles, we applied a “significance smoothing” to the gene expression data. This means, for each gene and for each pair of age stages (day 6 to day 9, day 9 to day 10, day 11 to day 12, day 12 to day 13, day 13 to day 14) the p-value indicates whether the corresponding gene is differentially expressed. If this p-value is less or equal than 1e-3, we assumed a significant differential expression for the corresponding gene during the two age stages and kept the original expression strength. If the p-value is above that threshold, we assumed no significant differential gene expression and correct the corresponding expression value for this age stage, using the mean expression value of the previous and current non-significant expression values (Figure S1 in File S1). After this smoothing step only significant expression patterns remained. Those patterns, which were very likely due to experimental or biological fluctuations, were corrected and smoothed. This led to expression profiles with no expression changes, which were removed from the dataset.

### Fuzzy Cluster Analysis

We performed a fuzzy cluster analysis to identify significant and striking age-dependent expression patterns generated during the lifespan of *P. anserina*. In contrast to hard clustering approaches, where disjunctive clusters are generated, fuzzy clustering leads to overlapping clusters. In this case, each profile belongs to each cluster with a given degree of membership in the interval [0; 1] where 1 indicates a total fit of the expression profile to the corresponding cluster core. Because gene expression itself and the experimental conditions as well can vary, this method is suitable as it avoids too stringent selection criteria. In our analysis we used the fuzzy clustering algorithm fuzzy c-means (FCM) [35] based on the modified R package “Mfuzz” [20]. The optimal number of clusters, which had to be defined *a priori*, was determined by means of the Xie-Beni index which computes a value for a fuzzy cluster result [36]. The smaller this index, the better is the partitioning. A repeated approach revealed an optimal size of eight clusters (Figure S2 in File S1).

## Results and Discussion

### Overview, Data Preparation and Validation

From single gene analyses it is known that aging of *P. anserina* is correlated with differential gene expression. In the corresponding analyses, transcript levels of selected genes (e.g., *PaDnm1, PaCtr3, PaSod2, PaMt1*) were found to differ in juvenile and senescent cultures [37]–[39]. Recently, we reported a differential genome-wide transcriptome analysis of juvenile strains of the long-lived mutant grisea and the wild-type ‘s’ [22]. This analysis allowed the assignment of 9,700 transcripts.

#### Age-related transcriptomes

In order to obtain a systematic view about age-related gene expression of this aging model, we performed a genome-wide longitudinal expression profile study of the wild-type strain ‘s’ [5]. In this analysis, total RNA was isolated from three independent individuals (biological replicates) after 6, 9, 10, 11, 12, 13 and 14 days of growth, pooled and subjected to a SuperSAGE analysis. Overall, more than 85 million single tag molecules were sequenced (Table S3) leading to 646,000 distinct different tags. A BLAST search led to the assignment of about 150,000 sequence tags to more than 10,200 of the predicted 10,635 genes encoded in the genome of *P. anserina*. After applying some filtering routines (e.g., removal of incomplete profiles) a “basic expression profile library” of 10,059 genes remained which covers about 95% of all genes in the genome of *P. anserina*. The processed expression data are compiled in Table S4.

#### Data validation

**Figure 1:**
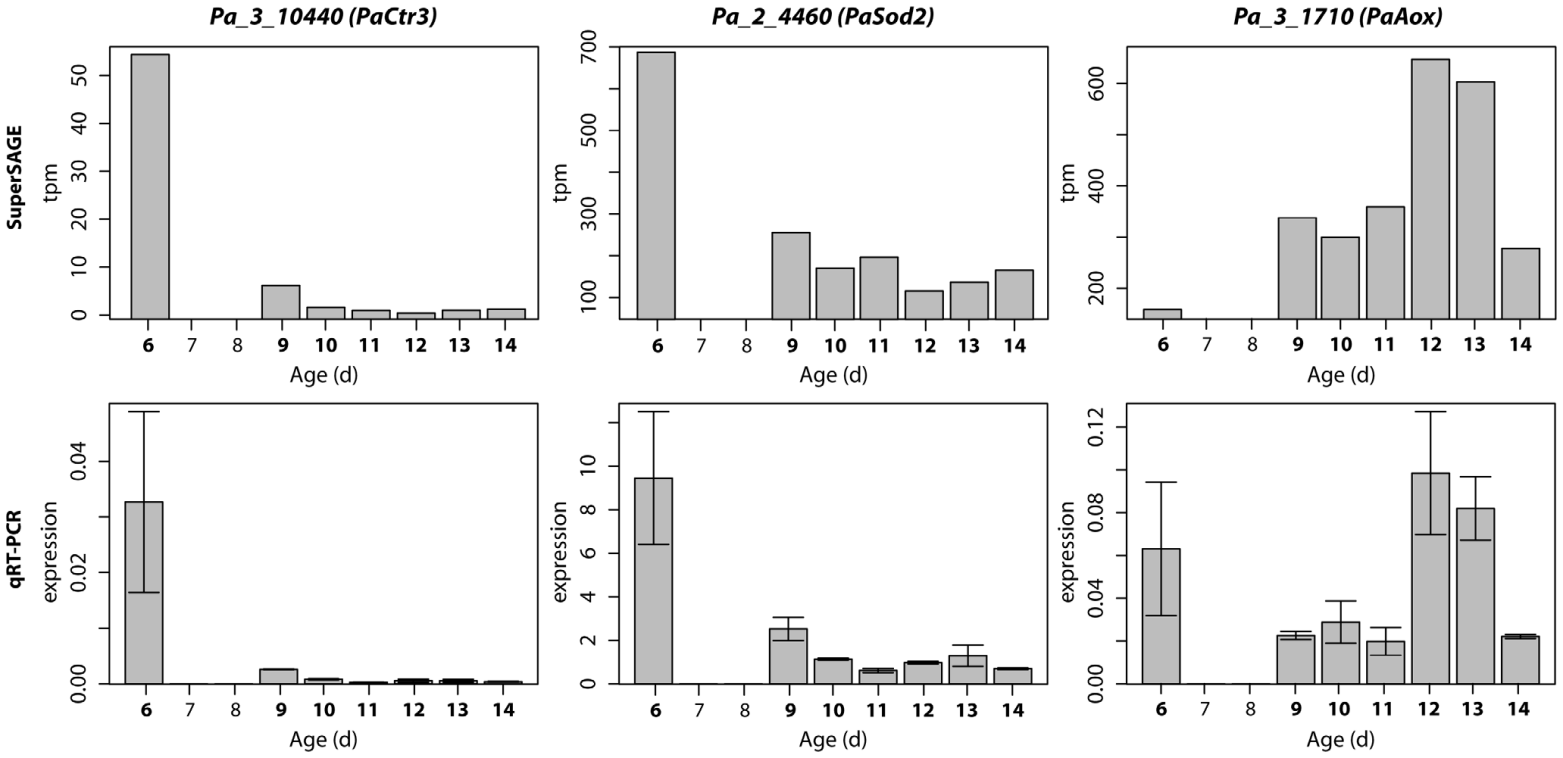
Transcript analysis of Pa_3_10440 (PaCtr3), Pa_2_4460 (PaSod2) and Pa_3_1710 (PaAox). Upper row: RNA of the three biological replicates (individuals) was isolated, pooled and analyzed by SuperSAGE. Gene expression was quantified as “tags per million” (tpm). Lower row: The same RNA was used for qRT-PCR analysis. RNA samples from each individual were individually analyzed for relative gene expression. Error bars represent the standard error. In both analyses, x-axes indicate the age of the individuals at which total RNA was isolated.

To validate the results obtained in the SuperSAGE analysis, qRT-PCR experiments were performed with three selected genes (*PaCtr3*, *PaSod2*, *PaAox*) of *P. anserina* (Figure 1). For *PaCtr3* and *PaSod2* it is known from previous work that their abundance changes from juvenile to senescent [38]. For *PaAox*, a northern blot analysis of the juvenile and the senescent stage revealed that the transcript was shown to decline during aging [40]. However, with the additional samples covering more age stages now, it becomes clear that the trend has a peak at days 12 and 13 (pre-senescent stage) and then declines in the oldest age stage (senescent, day 14). This trend has not been seen before when RNA samples from only two age stages juvenile and senescent were studied [40]. The increased expression of *PaAox* in the pre-senescent phase may be explained as a compensatory attempt to rescue the breakdown of cytochrome c oxidase dependent respiration.

The RNA samples of the current study were the same in both, the qRT-PCR and the SuperSAGE analysis. However, in contrast to the SuperSAGE study, the three samples (biological replicates) of each age stage were not pooled for the qRT-PCR, but analyzed separately, allowing the determination of a standard error among the different biological replicates. Comparing the highest and lowest transcript levels in each profile, at least 3-fold differences in RNA abundance were found for each gene. Moreover, the transcript levels determined by the two different techniques were comparable (Figure 1). This conclusion is also in agreement with another recent transcriptome analysis [22].

#### Gene Ontology (GO) term assignment

Using the *P. anserina* genome data base, we first performed an extended homology search to identify putative homologs encoded by all of the 10,635 putative *P. anserina* genes. We used this information to assign 2,334 additional proteins with an E-value of ≤1e-20. Overall, 7,609 (72%) of the 10,635 gene products are now assigned to GO terms (Table S2), while 2,451 of the genes in the basic expression profile library remain to code for putative products of unknown function.

#### Significance smoothing

In order to analyze the 10,059 expression profiles for age-dependent expression patterns and/or to investigate co-regulated genes associated with similar pathways and processes, we performed a cluster analysis using a Euclidean metric. For this purpose, we first standardized the 10,059 generated profiles to a mean of zero and a standard deviation of 1 (see: Methods). A major drawback of standardization approaches is that information regarding variances and dispersion become lost. For example, if a gene’s expression changes from 1 tag per million (tpm) to 3 tpm after standardization it seems to have the same variation as a gene’s expression change of 100 tpm to 300 tpm, even if these small expression changes (1 tpm to 3 tpm) are probably due to experimental noises. Hence, to avoid an over-fitting and over-interpretation, we first applied a “significance smoothing” to the 10,059 expression profiles and ended up with a “cluster profile library” of 7,467 reliable expression patterns which exhibit significant (age-related) differential expression.

### Fuzzy Cluster Analysis

In order to analyze the 7,467 profiles in the “cluster profile library” for age-dependent expression patterns and/or to investigate co-regulated genes associated with similar pathways and processes, we performed a fuzzy cluster analysis. We used the fuzzy c-means algorithm (FCM). For the results see Table S5. The optimal size of clusters was statistically determined to eight (see: Methods).

**Figure 2:**
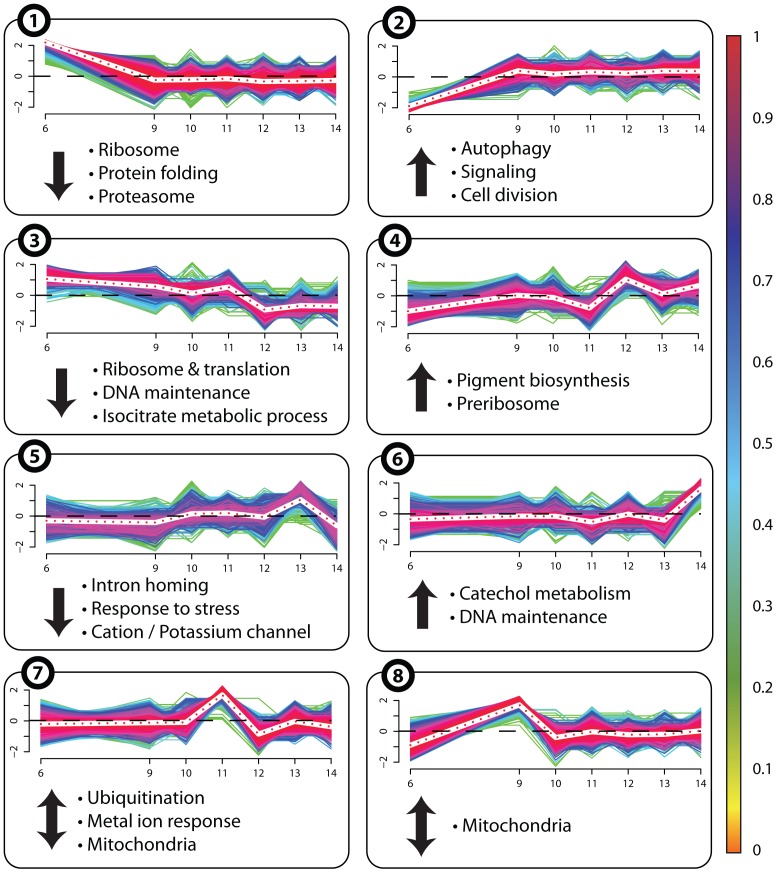
Fuzzy clustering analysis. All of the 7,467 “smoothed” expression profiles of the “cluster profile library” are distributed to eight fuzzy clusters. The x-axis represents the seven time points at which RNA was isolated from the three investigated *P. anserina* individuals. The y-axis indicates the relative expression strength. The color gradient corresponds to the membership value (see color bar on the right). A membership value of 1 means a perfect fitting to the corresponding cluster core (deep red). Arrows indicate the general tendency of the corresponding cluster. Below each cluster, a selection of categories to which significantly enriched GO terms (p-value ≤0.01) for particular functions were associated are listed (see description in the text).

Figure 2 depicts the eight fuzzy clusters and their corresponding expression patterns, including all of the age-dependent profiles of the *P. anserina* “cluster profile library”. In contrast to “hard” clustering approaches each profile fits to each “fuzzy” cluster with a specific membership degree. The thick white profile represents the core profile of each cluster and corresponds to the weighted mean of all profiles assigned. The color gradient used for the profiles indicates the congruence with the cluster core, i.e., a red profile displays a perfect fit to the corresponding cluster core profile (see color bar).

The eight main patterns, can be assigned to following profile types: (i) profiles showing a general decrease of transcripts (cluster 1, 3, and 5), (ii) profiles with a general transcript increase (cluster 2, 4, and 6), and (iii) profiles with a strong transcript increase earlier or later in the life cycle which subsequently falls back to the basic level (clusters 7 and 8).

In order to assign characteristic pathways, processes and components to the different clusters, we performed a GO enrichment analysis. The results of the complete analysis are provided in the supporting data (Table S6). In Figure 2, below each cluster, a selection of categories related to GO terms with particular interest for aging and lifespan control is indicated. The categories were manually generated by compiling entries from the GO universe. For instance, the terms “ribosome”, “small ribosomal subunit”, “cytosolic small ribosomal subunit”, “structural constituent of ribosome” and some more were grouped into the category “ribosome”.

Clusters 1 and 3 with down-regulated transcripts were found to contain enriched genes coding for products associated with protein folding (e.g., “peptidyl-prolyl cis-trans isomerase activity”), the “proteasome”, the “ribosome”, “DNA maintenance”, and the “isocitrate metabolic process”.

The main profile of cluster 5 describes a weak increase of expression till day 13 followed by a rapid decrease below the basal level. In this cluster, GO terms related to categories like “response to stress” and “cation/potassium channels” were found to be significantly enriched. While “response to stress” is a well characterized type of age-related processes basically nothing is known about the impact of “cation/potassium channels” in aging of *P. anserina*. In contrast, an important role of “intron homing“, another GO term identified to be enriched in cluster 5, has been well investigated before. ”Intron homing” was reported to be involved in the age-related reorganization of the mitochondrial DNA (mtDNA) as it occurs during aging of cultures [41]–[44]. The underlying mechanism is related to the mobility of the first cytochrome c oxidase intron [45], its release from and its reintegration into “homing sites” in the mtDNA and the subsequent recombination between intron repetition sequences [1], [44]. As a consequence, large parts of the mtDNA are deleted in senescent cultures leading to deficiencies in remodeling of affected proteins encoded by the mt genome.

Overall, stress response pathways are identified as being down-regulated during aging of *P. anserina*. This category includes the GO term “isocitrate metabolic process”, the role of which in regulating cellular defence against oxidative stress has been described before [46]. The other categories include DNA and protein quality control pathways like “telomere maintenance”, “recombinational repair”, “protein refolding”, protein degradation via the “proteasome”, and the replacement of affected proteins via the expression of the corresponding genes.

In cluster 2, in which transcripts increase in abundance at early life stages, enriched GO terms like “signal transduction”, “regulation”, “regulation of cell communication” or “intracellular protein kinase cascade” were identified and compiled in the category “signaling”. This category makes clear that during aging, there is a strong need for cellular readjustment and remodeling. This conclusion is in concordance with the down-regulation of important components (e.g., the proteasome) and the accumulation of impairments during aging [47]–[49]. A second enriched category of GO terms is associated with “cell division” and suggests an increased need for the expression of genes coding for products of the developmental machinery. Two additional GO terms, “autophagy” and “macroautophagy”, which are enriched in cluster 2, attracted our special interest. We combined these terms in the category “autophagy”. The up-regulation of genes in this category appears to be of special relevance in a situation where the proteasome as a major quality control component is impaired (see below).

In the second cluster with an up-regulated tendency (cluster 4), GO terms are found that are related to “pigment biogenesis”. In this category, GO terms, like “carotenoid metabolic process” and “pigment metabolic process”, were found to be enriched. Strikingly, in an earlier study [50] it was shown that an over-expression of carotenoid associated genes leads to increased lifespan of *P. anserina*, probably by the ROS scavenging activity of this class of pigments. Furthermore, we found enriched GO terms related to other pigment metabolic processes. This is in concordance with an age-related increase in pigmentation of *P. anserina* cultures. Strikingly, although the category “ribosome” was found in clusters with a decrease of transcripts, we identified GO terms which can be assigned to the category “pre-ribosome” consisting of terms like “rRNA processing” and “pre-ribosome, large subunit precursor”. Since early processes of the ribosomal assembly occur in the nucleus before these pre-ribosomal structures are exported to the cytosol for final maturation [51], it is reasonable to assume that the early processes in ribosome assembly are not affected. The observed increase in expression of genes, encoding pre-ribosomal proteins, may be an unsuccessful cellular rescue attempt.

In the third cluster with an up-regulated tendency (cluster 6), the GO term “SWI/SNF complex” was found to be enriched. This protein complex is part of the nucleosome remodeling complex and is implicated in various processes like gene expression, nuclear organization, centromere function, and chromosomal stability [52]. It is thus part of a category defined as “DNA maintenance”. Significantly, in contrast to the corresponding GO terms identified in cluster 3, transcripts linked to this term were found to increase during aging. A second category of GO terms, termed “catechol metabolism”, with particular relevance to aging was found to be significantly enriched by GO terms like “catechol catabolic process, ortho-cleavage“, and “catechol-containing compound, catabolic process”. Previously, work on *P. anserina* unravelled a role of such polyphenols in aging. In particular, it was found that the o-methyltransferase PaMTH1 as a longevity assurance factor, which increases in abundance during aging of *P. anserina* [53]–[55], appears to be involved in methylation of polyphenols with vicinal hydroxyl groups which, in the presence of Cu2+ and Fe3+, are prone to the generation of the highly reactive hydroxyl radical with its potential to damage all kinds of molecules [56]–[58]. The enzyme was shown to methylate various flavonoids (e.g., quercetin, myricetin) *in vitro*. Over-expression of the corresponding gene in *P. anserina* resulted in a reduction of protein damage and a lifespan extension [59]. The transcriptome data of the current study provide a first direct evidence for an age-related increase of enzymes associated with catechol in *P. anserina* (Pa_1_20370 and Pa_7_9460– “Put. hydroxyquinol 1,2-dioxygenase”), providing first evidence about *in vivo* substrates of PaMTH1. Significantly, this metabolism and the activity of the mammalian PaMTH1 homolog catechol-o-methyl-transferase (COMT) play a key role in a variety of human disorders including the age-associated Parkinson disease [60], [61].

In the clusters 7 and 8 transcript abundance increases transiently at different age stages and subsequently falls back to base line levels. These are the only clusters, which contain significantly enriched GO terms associated with “mitochondria” identified in the cluster analysis. Transcript levels increase from day 10 to 11 (cluster 7) and day 6 to 9 (cluster 8). At this period of time in the life cycle of the fungus, an optimal function of mitochondria to generate enough adenosine triphosphate (ATP) appears to be important for the generation of sexual reproduction structures (protoperithecia and spermogonia). In cluster 7, GO terms linked to the categories “ubiquitination” and “metal ion response” are enriched. Changes in metal ions concentrations in cellular compartments have been indeed demonstrated to occur during aging. In particular, an increase of cytoplasmic copper in earlier age stages has been observed during aging in *P. anserina* and human fibroblasts [38], [62]. These data are consistent with the observation of increases in the expression of controlling metal homeostasis (see below) in cluster 7. Finally, an increase in “ubiquitination” may be required for the induction of protein quality control pathways to control cellular proteostasis. The corresponding pathways in *P. anserina* have not been investigated yet.

### Continuously Up- or Down-regulated Expression Profiles

**Figure 3:**
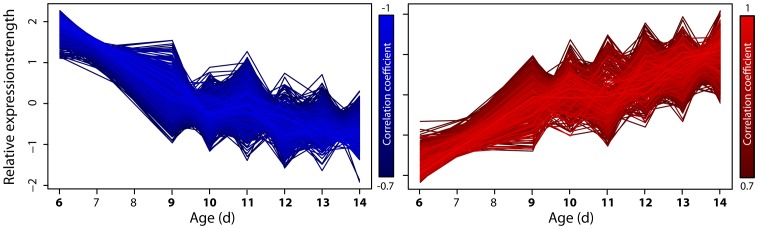
Age-related profiles of continuously down- or up-regulated genes. The Pearson correlation coefficient for each profile was determined. It indicates whether a gene was down- (≤−0.7) or up-regulated (≥0.7) during aging. The identified groups of down- (left) and up-regulated (right) expression profiles are depicted. The brighter (weaker) the color of a profile, the higher (lower) is the correlation coefficient (see color bars). Days on the x-axis in bold identify the time points, at which RNA samples were isolated. 1,202 genes with a decreasing (left) and 418 genes with an increasing tendency (right) were identified.

The cluster analysis was performed as a first rough and non-biased evaluation of all age-related expression profiles (“cluster profile library”) prepared for our bioinformatical analyses. Next, in order to strengthen the clues revealed by this analysis, we reduced the complexity of the data set to profiles exhibiting a continuously up- or down-regulation of gene expression (see: methods). We identified 537 genes displaying an increase and 1,766 genes with a decrease in abundance. In order to reduce the risk that the observed profiles result from experimental or biological fluctuation, we subsequently considered only those profiles with p-values less or equal to 1e-10 for differential expression between day 6 and 14 (first and last day in the measurement). The corresponding data libraries contain 418 genes with an up-regulation (right site of Figure 3), and 1,202 genes with down-regulated age-related trend (left site of Figure 3, see also Table S7).

**Figure 4:**
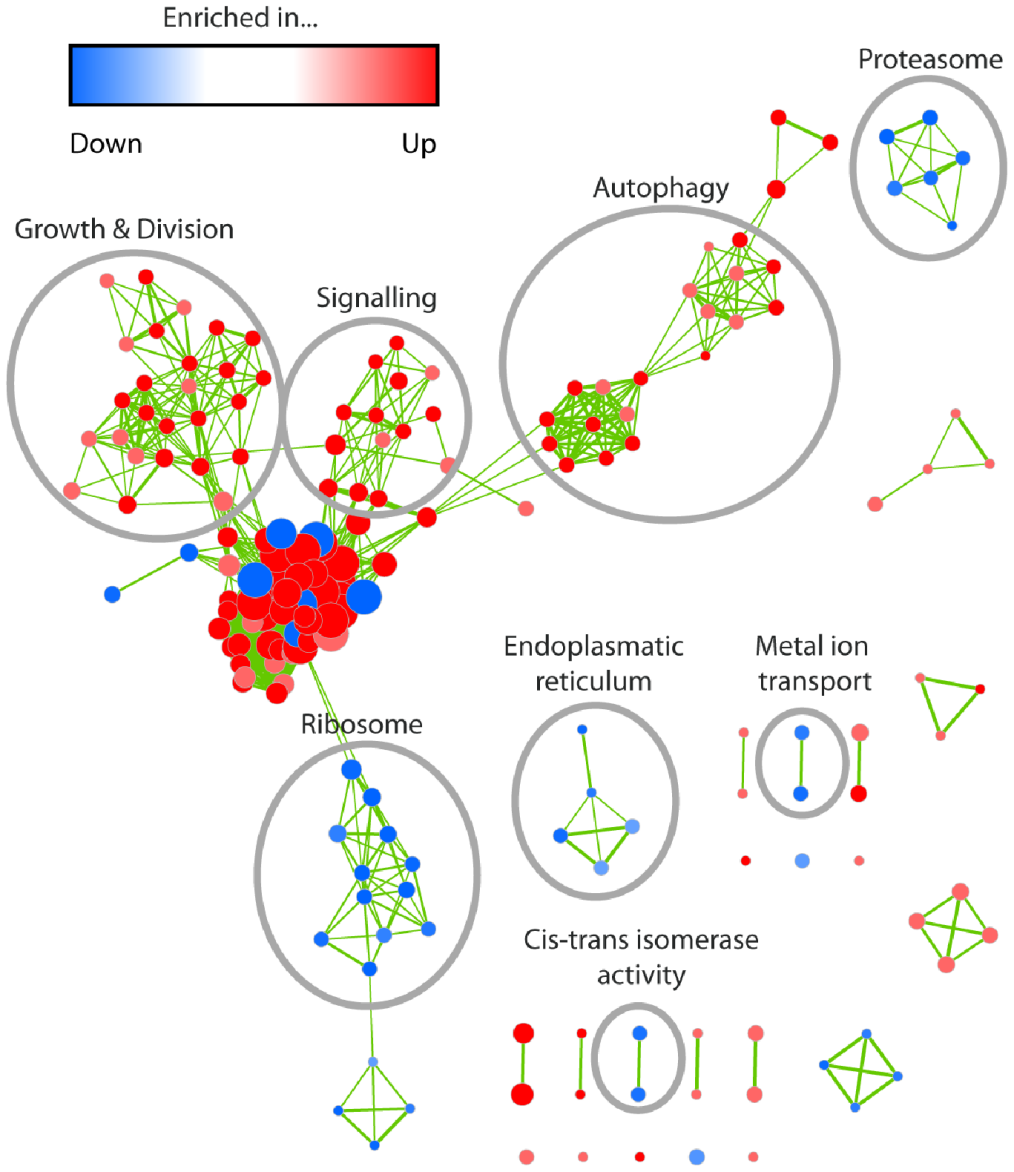
GO term enrichment map for the library of down- and up-regulated genes. Each node represents one GO term. The thickness of the edges represents the number of genes shared by two terms. The node color corresponds to the group and the degree of significance for enrichment (see color bar). Node sizes indicate the number of genes within the corresponding GO term. GO terms are grouped by their similarity degree defined by the number of common genes or related terminology. Striking groups were manually circled and labelled. Map regions with a high density of thick-sized nodes (center of figure) represent GO terms, describing very general processes with rather low information.

As in the cluster analysis, we performed a GO enrichment analysis using the two age-specific expression profile groups (Table S8). The reduction of the complexity of data allowed us to construct intuitive enrichment maps using the method introduced by [31] (see: Methods). Figure 4 represents an example for such a map. In this map each significantly enriched GO term (p-value ≤0.01) from both profile groups is depicted as a single node. The number of different genes in one node is indicated by the node size. Nodes that are sharing genes are connected by edges. The thickness of each edge corresponds to the number of genes shared by two nodes. GO terms enriched within the group of down-regulated genes are indicated in blue and those enriched in the up-regulated set in red. Nodes associated with similar categories of pathways and processes were manually circled. GO terms describing very general processes like “cellular metabolic process”, “intracellular” or “protein binding”, although they contain a larger number of genes, are not further investigated because they are linked to rather unspecific processes.

**Figure 5:**
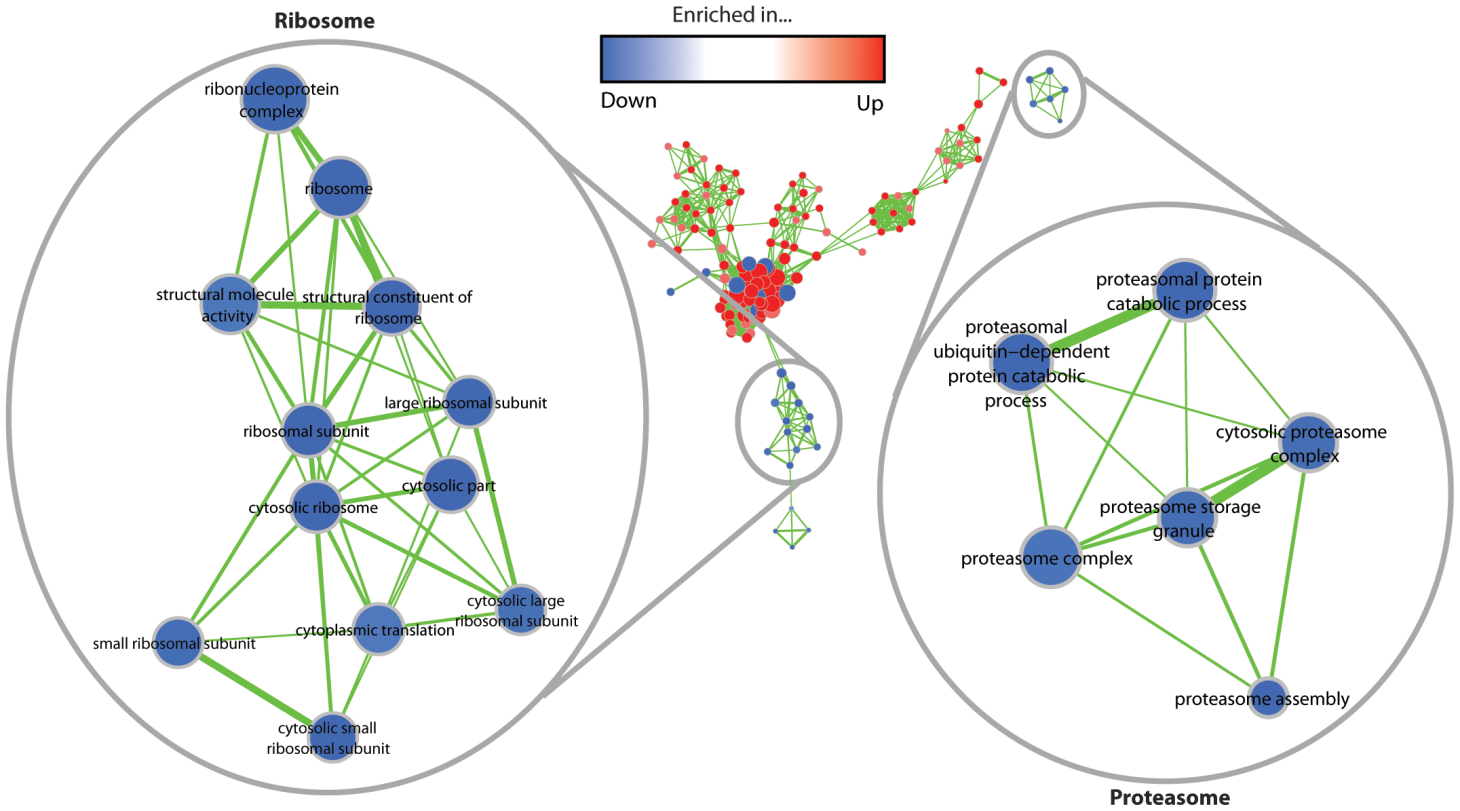
GO term enrichment map for the “ribosome” and the “proteasome” categories in the set of down-regulated genes. As in Figure 4, each node represents one GO term and node colors correspond to the group and the degree of significance. Edge thickness indicates the amount of genes shared by two terms and the node sizes are proportional to the number of genes assigned to the corresponding term.

As indicated in the cluster analysis before, the GO enrichment analysis revealed a significant enrichment of terms or genes associated with the ribosome and the proteasome (Figure 5). Most significantly, as revealed by a BLAST search, the GO term “proteasome assembly” contains the genes *Pa_2_8950* and *Pa_1_12250*, which encode two putative homologs of the human proteasome subunits beta 2 and 5, respectively. These subunits are also known to decrease in abundance in late passages of human WI38 fibroblasts along with beta subunits 1, leading to a reduced proteasome activity. Significantly, the over-expression of the genes, encoding either the beta subunits 1 or 5, can rescue proteasome activity [63].

Also an enrichment of components linked to the ribosome was found in the down-regulated clusters (Figure 5). These data are consistent with experimental findings reporting a decrease of ribosome-related transcripts during replicative aging in *Saccharomyces cerevisiae* [64]. The decrease may result from the deactivation of the “target of rapamycin” (TOR) kinase, a protein complex which is highly conserved from yeast to mammals and has also been studied in *P. anserina* [65]. This kinase is the central regulator of the TOR pathway [66], which is known to positively regulate the transcription of genes associated with ribosome biogenesis [67].

**Figure 6:**
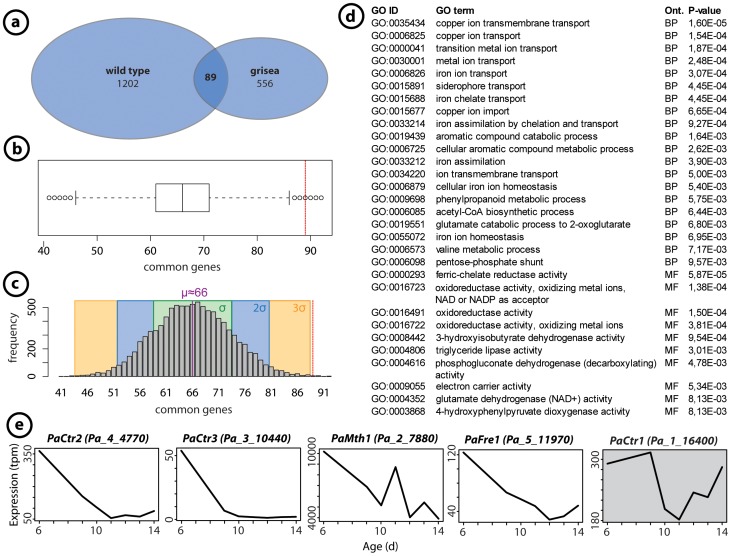
Comparison of down-regulated genes in the grisea mutant and the wild type. (a) Common down-regulated genes within the age-dependent transcriptome of the wild-type ‘s’ and the long-lived mutant grisea of *P. anserina*. 556 genes of the grisea transcriptome were identified to be down-regulated with a factor of at least 3 and a p-value of ≤0.01 for differential expression [22]. The 1,202 genes, identified as being down-regulated during aging in this study, have 89 genes in common. (b) 10,000 simulated experiments with two sets of randomly picked gene names, each set of the same size as the two sets (wild type, grisea), were applied. The box plot shows that the 89 common genes were extreme outliers. (c) The results of the random experiments are normally distributed with a mean of μ≈66.05 and a standard deviation of σ≈7.39. Our determined value of 89 is greater than three times sigma, indicating that the corresponding genes do not occur randomly. (d) All enriched GO terms in the set of the 89 common genes with a p-value ≤0.01 are depicted. Copper associated terms can be found on the very top. (e) Four genes already identified as putative target genes of transcription factor GRISEA are found within these 89 common genes. Due to its irregular expression profile, the gene, encoding the putative copper transporter PaCTR1, is not among the 89 common genes.

Among the GO terms which attracted our special interest were the terms “metal ion transport” and “cis-trans isomerases” (Figure 4). In the first category, the gene encoding the high affinity copper transporter CTR3 was found, which is regulated by the copper-sensing and -binding transcription factor GRISEA [68], [69]. From earlier work we know that transcription of *PaCtr3* strongly decreases during aging [70] as the result of an age-related increase in cytoplasmic copper and the repression of transcription factor GRISEA [62]. Apart from *PaCtr3*, genes encoding the two additional copper transporters, PaCTR1 and PaCTR2, the genes *PaMth1* encoding an o-methyltransferase, and *PaFre1*, which codes for a putative ferric reductase, were suggested as potential target genes of GRISEA [22]. In order to validate this conclusion and to identify additional putative genes regulated by transcription factor GRISEA, we compared the 1,202 down-regulated genes from the current age-related study with the 556 down-regulated genes in the grisea mutant (Figure 6a). We found 89 genes to overlap in the two datasets (Table S9). In order to test, whether the number of overlapping genes is higher than expected by chance, we randomly draw from the pool of the 10,059 available genes of the wild-type transcriptome 1,202 genes and subsequently from the 9,704 transcripts of the ‘grisea’ genome 556 samples. This procedure was performed 10,000 times. The results are compiled in the box plot shown in Figure 6b. The red line indicates that the 89 identified genes lay beyond the upper whisker of the box plot and are extreme outliers. As expected, the results of this random experiment are normally distributed (Figure 6c) with a mean of μ≈66.05 and a standard deviation of σ≈7.39. Regarding our observation of 89 common genes, 89≥3σ holds, indicating that the identification of 89 common genes is very unlikely to occur by chance.

Next, we applied a GO analysis for this set of 89 genes. The most significantly enriched GO terms are associated with the copper metabolism (Figure 6d). While *PaCtr2*, *PaCtr3*, *PaFre1*, and *PaMth1* were found within the overlapping genes, *PaCtr1* was not found in this group (Figure 6e). Comparing the age-related transcript profiles of the five genes revealed that the profile of *PaCtr1* differs strongly from those of the four other genes. After a decrease of transcripts in the middle of the life cycle there is an increase late in life, indicating that the regulation of this gene may not exclusively be controlled by transcription factor GRISEA and cytoplasmic copper. Also the profile of PaMth1 with an increase of transcripts may be influenced by additional regulatory circuits which remain to be elucidated.

In our analysis, the down-regulation of genes, coding for cis-trans isomerases, was rather surprising, since earlier experiments indicated an increase during aging of cyclophilin D (PaCYPD), a mitochondrial enzyme of this group [54]. Moreover, this regulator of the mitochondrial permeability transition pore was identified to induce a cell death program at the end of the life of *P. anserina* [71]. A close look to the individual genes grouped in the list of GO terms revealed that the gene coding for PaCYPD was not included. The transcription profile of this gene shows that at early life stages the transcript exhibits a fluctuating profile with a strong increase in expression at the last age stages (days 13 and 14).

One significantly enriched group of GO terms within the up-regulated gene set is related to “signaling”. Here, various components, including kinases and phosphatases and known components of signal transduction pathways, were identified (Table S8). As the GO terms on “regulation” are also significantly enriched, it becomes clear that during aging there is a strong need to re-adjust the cellular metabolism. This need is in concordance with the down-regulation of important components (e.g., the proteasome) and the accumulation of impairments during aging.

**Figure 7:**
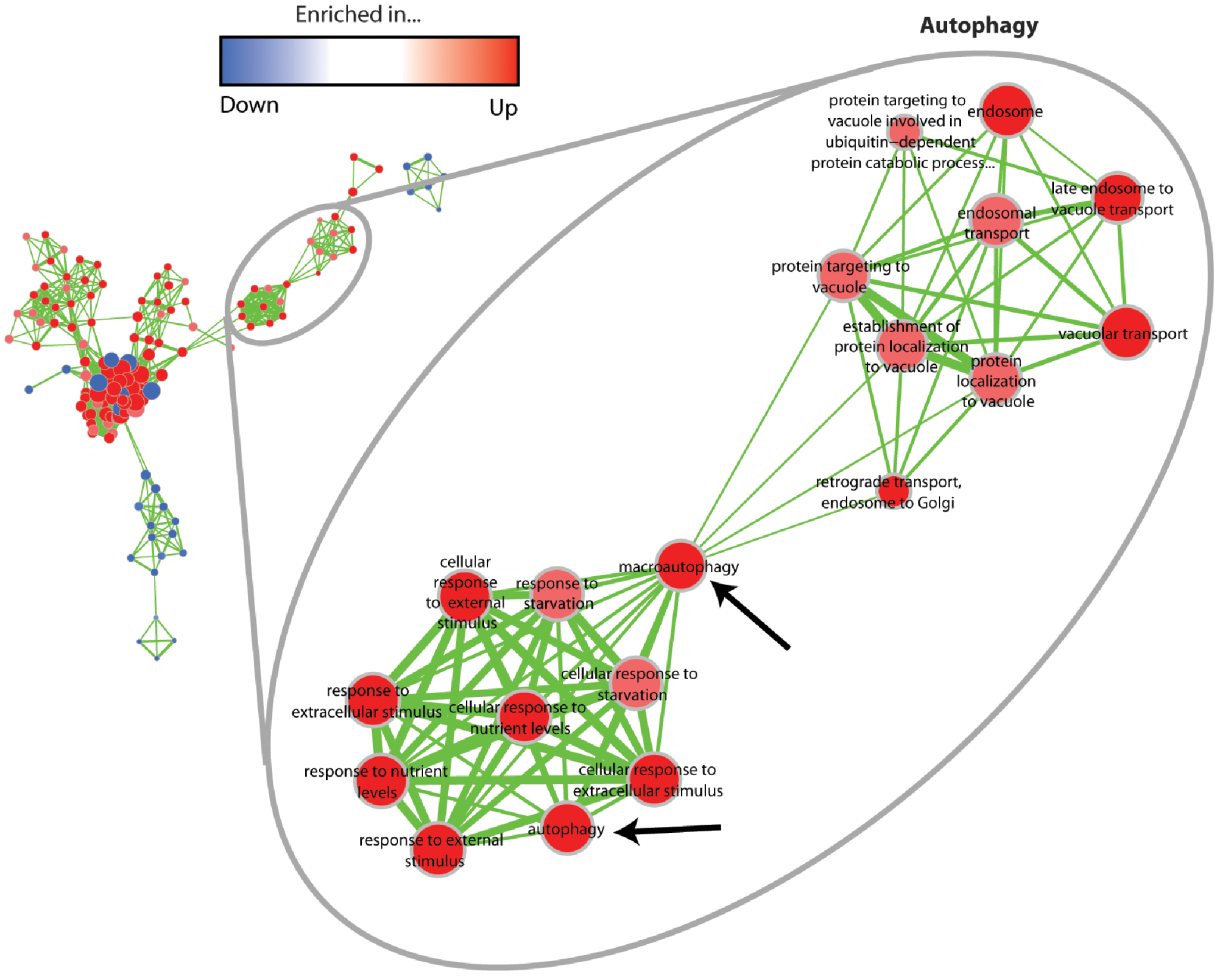
GO term enrichment map for the “autophagy” category in the up-regulated gene set. Analogous to Figure 4, each node represents one GO term and node colors correspond to the group and the degree of significance. Edge thickness indicates the amount of genes shared by two terms, and node sizes are proportional to the number of genes assigned to the corresponding term.

While the proteasome as a component of cellular quality control systems appears to be down-regulated during aging, another cellular quality control pathway, macroautophagy (autophagy), becomes induced (Figure 7). This is indicated by the up-regulation of genes encoding homologs of ATG proteins, signaling components and proteins involved in ubiquitination. Specifically, we found that the abundance of the transcript of an ATG13 homolog, which is essentially for autophagy initiation, increases during aging. Apart from the transcripts coding for an ATG13 homolog, transcripts of ATG6 and VPS15 were identified in the group of up-regulated transcripts coding for putative components of the autophagosome. VPS15 is a serine/threonine kinase, which regulates the activity of VPS34. Both proteins act together in the class III phosphatidylinositol 3-kinase (PI3K) complex necessary for the nucleation and assembly of the initial phagophore membrane and have been shown to be essential in the filamentous fungus *Sordaria macrospora* [72]. On first glance, it is surprising that only *PaVps15,* but not *PaVps34,* is continuously up-regulated during aging. However, from the transcript data it is not clear, which one of the transcripts is limiting and how efficient the corresponding mRNAs are translated. Nevertheless, despite the general difficulties to conclude on the activity of specific proteins, an interesting new aspect arose from the transcriptome analysis.

Autophagy is negatively regulated by the TOR kinase which phosphorylates ATG13. Phosphorylated ATG13 is unable to initiate autophagy via the interaction with ATG1 to form the pre-autophagosomal structure [73]. Both, the transcriptional down-regulation of ribosome genes as well as the up-regulation of macroautophagy transcripts suggests that TOR becomes inactivated during aging of *P. anserina*. It seems that this inactivation acts as a compensatory mechanism to delay deleterious effects of the age-dependent damage accumulation, since TOR inhibition leads to lifespan extension [74]–[76]. However, obviously, this process is not able to prevent the organismal death finally, especially since in *P. anserina* during aging the mtDNA becomes grossly rearranged, leading to a complete loss of the remodeling capacity of mitochondria-encoded proteins [77]. Autophagy has been reported to occur in different filamentous fungi and is important for fungal development, sexual and asexual reproduction and pathogenicity (reviewed in [78], [79]). In a recent study, it has been described that autophagy promotes survival in ‘aging’ submerged cultures of *Aspergillus niger* [8]. It should be stressed that this kind of ‘aging’ is different from ‘organismic aging’ as a process that occurs under nutrient replete conditions. The decline of cellular functions during organismic aging is studied in various aging models (e.g., *P. anserina*, *Caenorhabditis elegans*, *Drosophila melanogaster*, mammals) and is not the result of starvation. Under nutrient starvation autophagy acts mainly as a recycling process to ensure cellular homeostasis even under conditions where the constant supply of nutrients is disturbed. Beside this important role of starvation-induced autophagy, autophagy has been demonstrated to have another important function. It serves as part of a complex quality control system involved in the removal of functionally impaired molecules and organelles (reviewed in: [80]–[82]). This latter function is of key importance to slow down aging of systems and thus has an important impact on lifespan.

The induction of autophagy as a major quality control pathway under conditions the function of the ubiquitin proteasome system (UPS) is impaired, is in agreement with earlier suggestions that autophagy is a compensatory mechanism, allowing the cell to remove UPS substrates. This conclusion is supported by investigations, using mouse cell lines, in which autophagy was induced by rapamycin and the proteasome inhibited by lactacystin. Under these conditions, lactacystin-induced apoptosis and levels of ubiquitinated protein aggregates were reduced, suggesting that autophagy induction acts as a compensatory pathway [83]. Similar conclusions were drawn in *Drosophila melanogaster* in which autophagy was induced as a result of mutations affecting the function of the proteasome [84]. In another study, Gamerdinger et al. [85] found two members of the BAG family, BAG1 and BAG3, to be key modulators of the proteasomal and autophagic pathways, respectively. While expression of *Bag1* becomes down-regulated during cellular and brain aging, *Bag3* expression is up-regulation in aged cells suggesting a molecular switch between autophagic and proteasomal degradation. In our study, we provide first evidence that autophagy becomes induced during normal organismal aging when the proteasome appears to be functionally impaired. Since ubiquitination is relevant for both, the degradation via the UPS system and for autophagy [86], it is not surprising that the GO term “ubiquitination” is not down-regulated like the GO term “proteasome”.

### Significantly Differential Expression between a Young and an Old Individual

**Figure 8:**
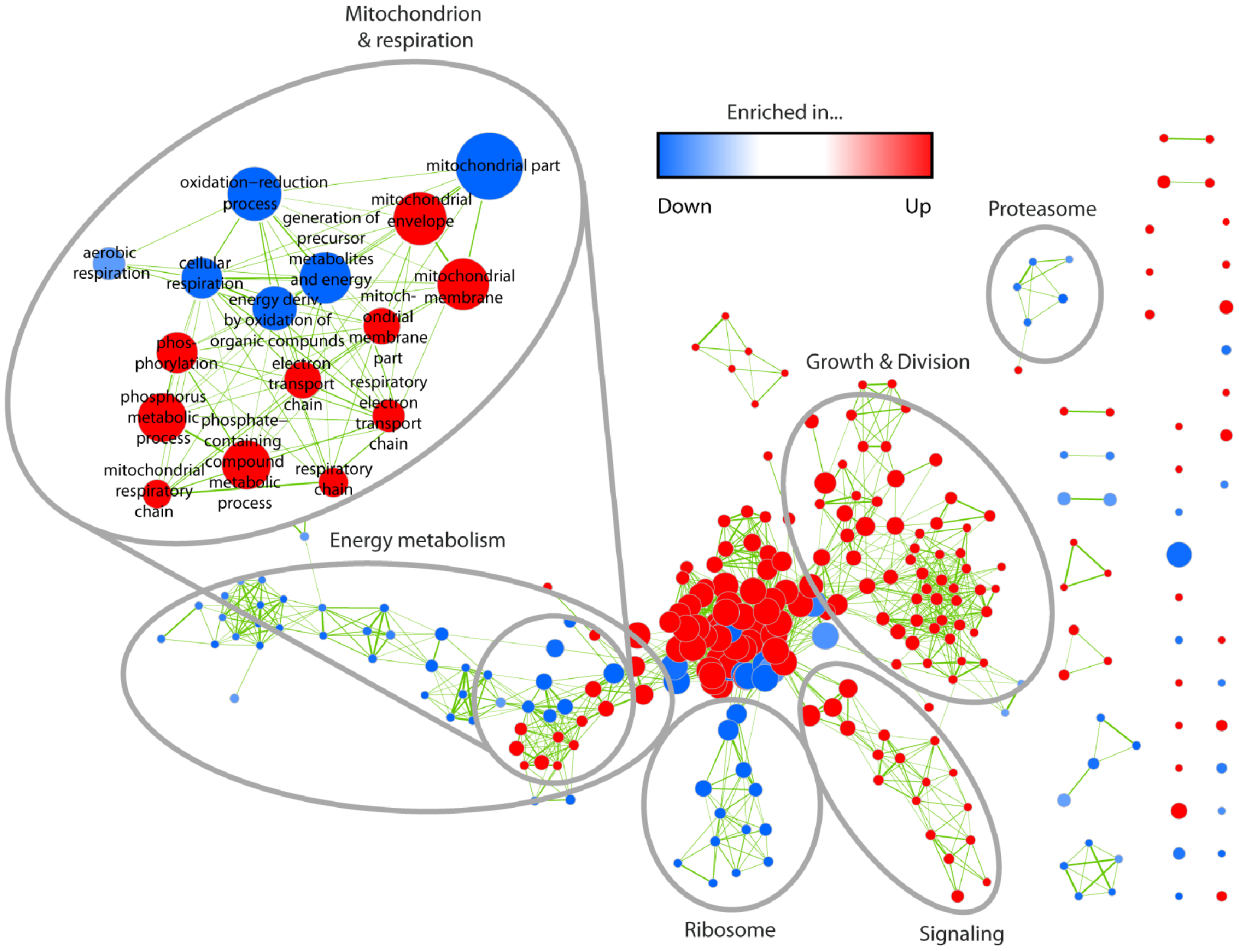
Transcriptome comparison of young (day 6) and old individuals (day 14). The figure depicts the enrichment map for the corresponding GO enrichment analysis. Analogous to Figure 4, each node represents one GO term and node colors correspond to the group and the degree of significance. Edge thickness indicates the amount of genes shared by two terms and node sizes are proportional to the number of genes assigned to the corresponding term. Striking groups were subsequently circled and labelled.

In a final comparative approach we reduced the transcriptome data sets by comparing only transcript levels in samples of the youngest (6 days) and oldest (14 day) individuals. Subsequently, the data were filtered for all p-values for differential expression of ≤1e-10 leading to 3,976 significantly differentially expressed genes. This gene set contains 2,037 up-regulated and 1,939 down-regulated genes (Table S10). The subsequent GO analysis (Table S11) shows GO terms which were not identified in the same clarity in the former analyses. In particular, genes associated with the energy metabolism, and especially the mitochondrial respiratory chain and the citric acid cycle are significantly enriched within both, the up- and the down-regulated group (Figure 8). The terms in the category “energy metabolism” specifically contains the GO terms “tricarboxylic acid cycle” and “pyruvate metabolic process” and a sub-category of “mitochondrion & respiration” with GO terms, like “mitochondrial part”, “respiratory chain”, or “cellular respiration”. Mitochondria and the energy metabolism are well-known to play a key role in aging of *P. anserina* and other organisms [87]–[90]. Here, it is of particular interest that only the comparative analyses of two age stages, young vs. old, led to the identification of this correlation which appears to vanish in the longitudinal transcription analysis when all age stages are evaluated. This effect may be due to the fact that the energy metabolism machinery is differentially regulated over the life cycle with fluctuations in gene expression at certain times, according to the energetic demands during development. Bioinformatical processing of these changes, e.g., by applying ‘fuzzy’ algorithms, may lead to the loss of important information. Applying different bioinformatics approaches in parallel may help to minimize the risk to miss important clues and conclusions.

## Conclusions

In this study, we report the results of a genome-wide longitudinal transcriptome analysis of the aging model *P. anserina*. Such an analysis is unique and cannot easily be performed in many other organisms, in particular in long-lived species. In our study, quantitative changes in the transcriptome were followed from an early age to a time-point shortly before the corresponding individuals die. The data represent a unique reference data resource that is valuable also for studies with other organisms. It becomes clear that the expression of genes over the lifetime of an organism does not just follow a simple up- or down-regulation. Many genes are differentially regulated at different times. A reliable comparison of data sets is therefore only possible if the correct samples from a multiple data set are analyzed. Failure in doing so may lead to contradicting results as they are often found in the literature.

The bioinformatical analysis of the transcriptome data in our study led to different relevant conclusions. For instance, we found that after having generated a complete data library it is helpful to reduce the complexity of this library to not miss important information about the processes studied. As a key conclusion of our study, we identified, for the first time, the induction of genes involved in autophagy during normal organismal aging. The induction of this quality control pathway was found to occur after the function of the proteasome and the ribosome is impaired, suggesting that autophagy induction may act as a compensatory pathway in situations when other quality control pathways fail. This is in concordance with earlier findings (reviewed in: [91]). Certainly, the conclusions from our analysis require a careful validation by next generation experiments. In *P. anserina*, such experiments can be performed, specifically by switching from high-throughput analysis to a specific genetic analysis. Experiments to demonstrate the significance of autophagy for aging and lifespan control of *P. anserina* and to elucidate the mechanisms triggering the observed responses have been raised. They will be published in a separate paper.

## Supporting Information

Table S1 Summary of primer sequences used in qRT-PCR analysis of selected genes.
(XLS)

Table S2 GO term annotation library of *P. anserine.*
(XLS)

Table S3 Counted tags for each day.
(XLS)

Table S4 Result of the transcriptome analysis.
(XLS)

Table S5 Fuzzy cluster analysis.
(XLS)

Table S6 GO enrichment of transcript profiles in the individual clusters.
(XLS)

Table S7 Compilation of continuously down- and up-regulated genes.
(XLS)

Table S8 GO enrichment analysis of the continuously up- and down-regulated *P. anserina* genes.
(XLS)

Table S9 Putative target genes of transcription factor GRISEA.
(XLS)

Table S10 Up- or down-regulated genes in RNA preparations from 6-day and 14-day old cultures.
(XLS)

Table S11 GO enrichment of up- and down-regulated genes in RNA samples from 6-day and 14-day old cultures.
(XLS)

Text S1 Tag-library correction and normalization to tags per million.
(DOCX)

File S1 Figure S1. Samples for the smoothing approach. Figure S2. Computed Xie-Beni indices for several cluster result.
(PDF)
