# Correction: A Genome-Wide Longitudinal Transcriptome Analysis of the Aging Model *Podospora anserina*

**DOI:** 10.1371/annotation/03280dea-66ce-4ba6-8ac5-f985f51dea37

**Published:** 2013-12-31

**Authors:** Oliver Philipp, Andrea Hamann, Jörg Servos, Alexandra Werner, Ina Koch, Heinz D. Osiewacz

The species name "*Podospora anserina*" is spelled incorrectly in the article title. The correct title is: "A Genome-Wide Longitudinal Transcriptome Analysis of the Aging Model *Podospora anserina*."

The correct Citation is: Philipp O, Hamann A, Servos J, Werner A, Koch I, et al. (2013) A Genome-Wide Longitudinal Transcriptome Analysis of the Aging Model *Podospora anserina*. PLoS ONE 8(12): e83109. doi:10.1371/journal.pone.0083109

