# Correction: A Genome-Wide Longitudinal Transcriptome Analysis of the Aging Model *Podospora anserina*

**DOI:** 10.1371/journal.pone.0091590

**Published:** 2014-03-25

**Authors:** The PLOS ONE Staff

In the Funding Statement, an organization providing funding to the last author (HDO) is incorrectly omitted. The Funding Statement should read:

"This work was supported by a grant by the Bundesministerium für Bildung und Forschung (BMBF; GerontoMitoSys 0315584A) and the LOEWE excellence initiative of the State of Hesse within the framework of the Cluster for Integrative Fungal Research (IPF) to HDO. The funders had no role in study design, data collection and analysis, decision to publish, or preparation of the manuscript."
