# Supplementary material for: A Genome-Wide Longitudinal Transcriptome Analysis of the Aging Model Podospora anserine
Source: PLoS One. 2013 Dec 20;8(12):e83109. doi: 10.1371/journal.pone.0083109 (PMC3869774; doi:10.1371/journal.pone.0083109)
Supplement: Text S1 — Tag-library correction and normalization to tags per million. (DOCX) [file pone.0083109.s012.docx]

**Supporting Text S1. Tag-library correction und normalization to tags per million.**

Due to the use of two anchoring enzymes (NlaIII and DpnII)), two restriction sites exists (CATG and GATC), which exhibit different ligation efficiencies. Furthermore, the tag library sizes of each sample differ in size. To handle these drawbacks a library correction was applied and subsequently the absolute expression values were normalized to tags per million for each sample. This was performed using the following steps:

1. Divide each sample in a set of CATG-tags and a set of GATC-tags.
2. To avoid outliers remove 1% of most expressed tags in both sets.
3. Compute the average number of each tag-species (average for CATG and for GATC) that was expressed (e.g. number of distinct CATG-tags in a sample / number of all CATG-tags in a sample).
4. Compute correction factor by: CATG-average / GATC-average.
5. Compute the corrected library size by: number of all CATG-tags + number of all GATC-tags * correction factor.
6. Correct each GATC-tag by multiplication of each tag with the specific correction factor of the corresponding library.
7. Convert each absolute tag count to tags per million (tpm): corrected absolute tag counts * 10^6 / corrected library sizes.
