# Supplementary material for: A Genome-Wide Longitudinal Transcriptome Analysis of the Aging Model Podospora anserine
Source: PLoS One. 2013 Dec 20;8(12):e83109. doi: 10.1371/journal.pone.0083109 (PMC3869774; doi:10.1371/journal.pone.0083109)
Supplement: File S1 — Figure S1. Samples for the smoothing approach. Figure S2. Computed Xie-Beni indices for several cluster result. (PDF) [file pone.0083109.s013.pdf]

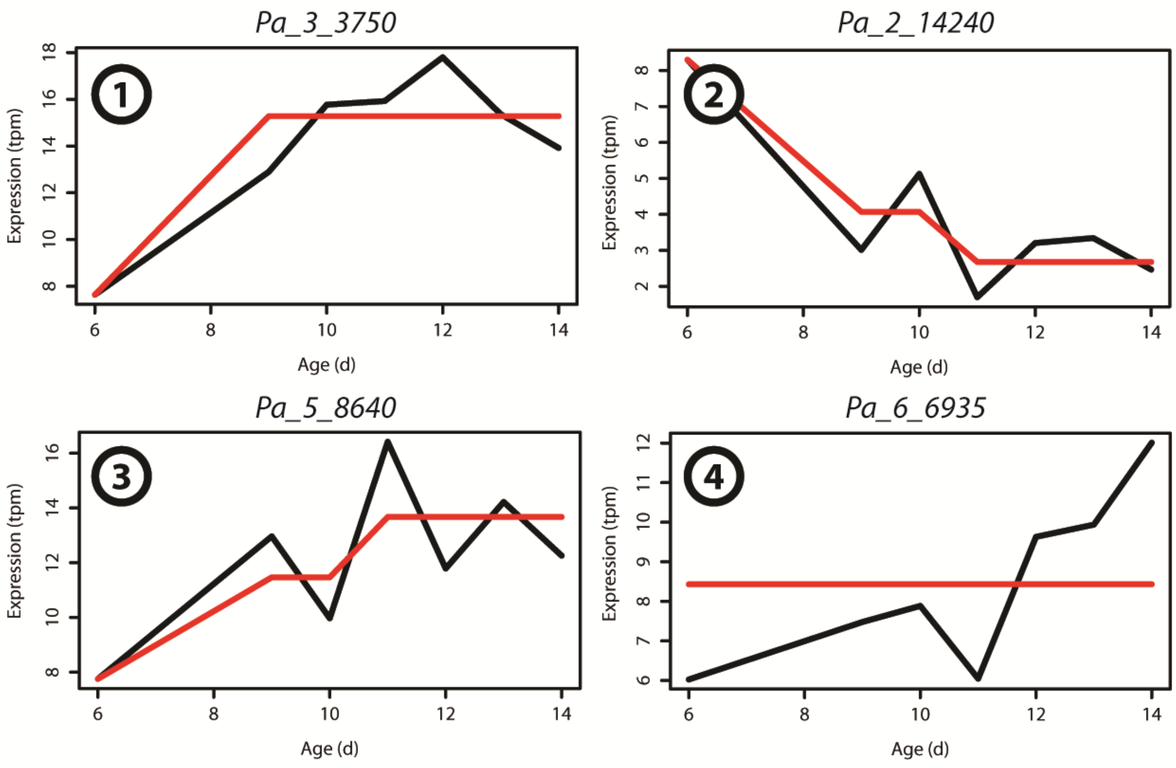

Supporting Figure S1. Samples for the smoothing approach. Each plot shows the original expression profile (black) and the corresponding significance smoothed profile (red), where each non-significant expression change has been removed (p-value ≤ 0.001). For example, sample 4 showing a completely non-significant expression pattern (no differential expression) is removed from the datasets for further analyses.

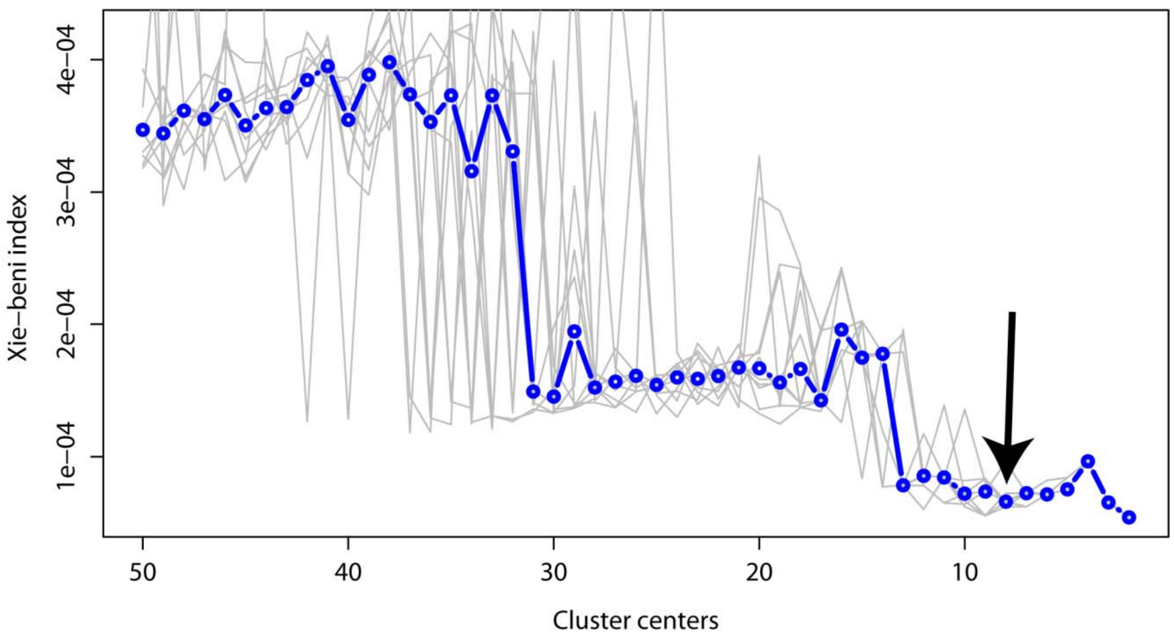

Supporting Figure S2. Computed Xie-Beni indices for several cluster results. Each gray graph represents one “cluster-run” with the Xie-Beni indices (y-axis) for clusters with 50 to 2 initial centers (x-axis). The blue graph represents the median graph of the 10 “cluster-runs”. The less the index, the best is the partitioning. Cluster sizes of 3 or 2 seem to result in lower indices, but led to an insufficient discrimination of the expression profiles. Therefore, a cluster size of eight has been chosen as best.
